# New distribution records of wild bees (Hymenoptera, Apoidea) in South Tyrol (Italy): expanding the wild bee database

**DOI:** 10.3897/BDJ.13.e138625

**Published:** 2025-02-25

**Authors:** Sebastiano Zanini, Matteo Dainese, Timo Kopf, Lisa Obwegs, Matteo Anderle, Georg Leitinger, Ulrike Tappeiner

**Affiliations:** 1 Universität Innsbruck, Innsbruck, Austria Universität Innsbruck Innsbruck Austria; 2 Eurac Research, Bolzano/Bozen, Italy Eurac Research Bolzano/Bozen Italy; 3 University of Verona, Verona, Italy University of Verona Verona Italy; 4 Freelance Biologist, Innsbruck, Austria Freelance Biologist Innsbruck Austria

**Keywords:** nature conservation, agroecosystem, biodiversity, Red List, mountain region

## Abstract

**Background:**

Throughout South Tyrol, in northern Italy, there is a data deficiency relating to wild bee species pool. Here, we present significant findings from the collection of 3,313 wild bees gathered over two separate studies conducted in consecutive years. Our research focused on the impact of landscape heterogeneity, temperature and land-use change on wild bee communities and their pollination services in an agricultural and mountainous landscape. This article provides a detailed account of the 150 identified wild bee species collected using coloured pan traps. We report habitat type, occurrence data, threat status, sociality, nesting strategy and diet breadth. In Italian regions where information on wild bee distribution is lacking or outdated, sharing data is crucial for developing conservation policies.

**New information:**

The compiled species list strengthens regional and national wild bee database by providing new distribution data for extinction-threatened species, such as *Dufoureadentiventris* (Nylander, 1848), *Dufoureainermis* (Nylander, 1848), *Lasioglossumbrevicorne* (Schenck, 1870), *Lasioglossumlaevigatum* (Kirby, 1802), *Lasioglossummonstrificum* (Morawitz, 1891), *Nomadamutica* Morawitz, 1872 and *Nomadavillosa* Thomson, 1870. Additionally, we present recent findings of species that are valuable for understanding range expansions, recording species previously unreported in South Tyrol and updating historical data for the region.

## Introduction

Over the last 30 years, pollinator abundance and diversity have declined globally due to multiple factors, primarily anthropogenic pressures (Hallmann et al. 2017, Powney et al. 2019, Granata et al. 2023, Ulyshen and Horn 2023). Land-use change, intensive agricultural management alongside associated pesticide use, invasive alien species and climate change are the main threats to pollinators (Potts et al. 2016). The conservation of pollinators is of utmost importance as they play an essential role in the life cycle of many organisms, including humans. An estimated two-thirds of global food crops require pollination to set fruits and seeds (Klein et al. 2006). Animals pollinate 87.5% of crop and wild plant species (Ollerton et al. 2011) and from an economic perspective, the ecosystem service provided by pollinators amounts globally to approximately $351 billion (USD)/year for food production (Lautenbach et al. 2012). In Europe, considering horticulture alone, more than 4000 vegetable varieties depend on wild and managed bee pollinations (Kluser et al. 2010). Thus, the commitment to curb pollinator decline is pivotal to three of the Sustainable Development Goals (SDG) from the Food and Agriculture Organization of the United Nations (FAO 2018): ensure biodiversity (SDG 15), food security (SDG 2) and, ultimately, human well-being (SDG 1).

The European Commission supports pollinator projects through funding programmes. However, a significant data gap exists on wild bee species, especially in central-southern Europe (Quaranta et al. 2004). Italy, for example, is a hotspot for more than 1,000 species (Comba 2019, Reverté et al. 2023), but the first complete national catalogue of bee species was produced only in 1995 by Pagliano G. (Quaranta et al. 2018). Since then, research has made progress, but with unequal effort and continuity in every region (Quaranta et al. 2002). Sharing new distribution records is important to build a numerical reference that will contribute to assessing the demographic trends of wild bee populations objectively. Ultimately, the aim is to evaluate the status of wild bee species for conservation, but this is only possible once data are available (Quaranta et al. 2018).

Mountain regions are valuable study areas because the complex topography offers many niches for species adapted to various microclimates and habitats. The interplay of abiotic pressures along the elevational gradient allows species to co-exist with different ecological requirements, making valleys and mountainside ecosystems critical for biodiversity conservation (Körner 2002). With climate change, these highly dynamic areas face potential species migration from warmer to colder regions, including higher elevations (Körner 2003). Additionally, species may shift along the latitudinal gradient, adapting to different climatic conditions or finding new niches (Wasof et al. 2013).

In 2021 and 2022, we conducted two separate studies in the agricultural landscapes of South Tyrol, a mountainous region in northern Italy. These studies involved surveys in various habitats, including apple orchards (14), vineyards (5), pastures (4), meadows (6), orchard meadows (2) and annual crop fields (2). After identifying the wild bees caught with pan traps, we evaluated their IUCN Red List status for Europe (Nieto et al. 2014) and Italy (Quaranta et al. 2018). Since South Tyrol lacks a Red List of wild bee species, we compared our findings with the most recent and complete synopsis of wild bees in South Tyrol (Hellrigl 2006). This data paper aims to provide open access to wild bee abundance data and a list of species in a region of substantial ecological importance. These data expand the database on wild bees for South Tyrol.

## Materials and methods

**Figure 1.**
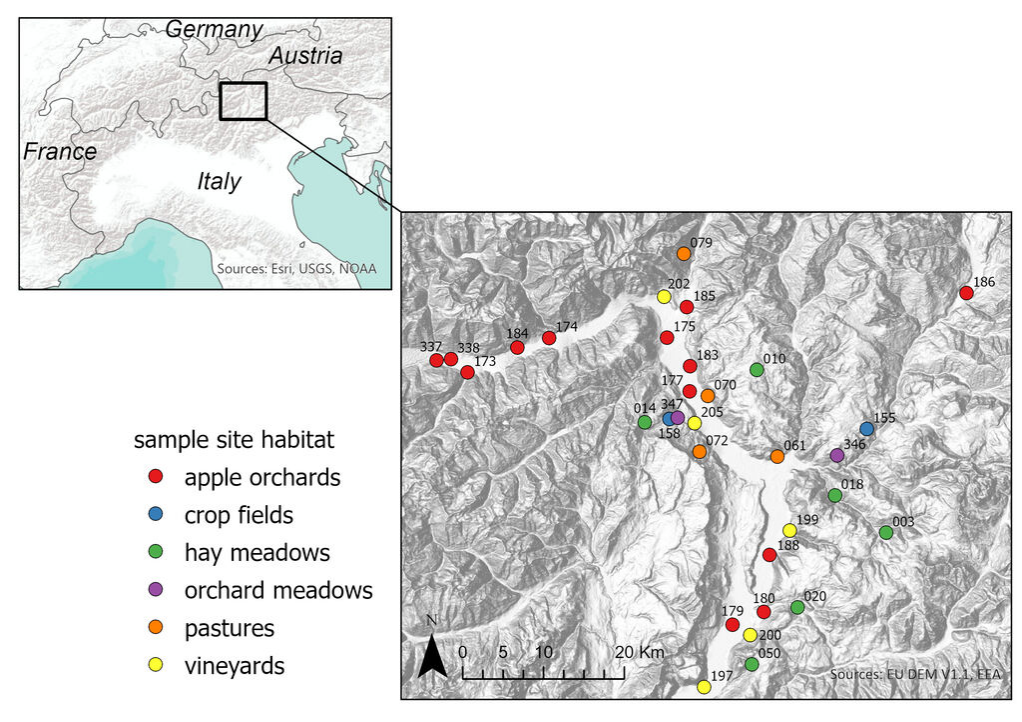
Section of the Autonomous Province of Bolzano/Bozen, Italy, with the study area and sites categorised by habitat class. Site code specifications and coordinates can be found in Suppl. material 1.

The Autonomous Province of Bolzano/Bozen - South Tyrol is dominated by mountains and is rich in forests, grasslands, watercourses and lakes. Valley floors are intensively cultivated and mainly occupied by settlements, managed apple monocultures and vineyards. With increasing elevation, cropland is progressively substituted by meadows, pastures and forests. Despite the intensively cultivated valleys, natural and semi-natural habitats (SNH) cover 82.3% of the region’s surface (Anderle et al. 2022). Data collection occurred within two valleys: Eisacktal/Valle Isarco (from Bozen/Bolzano to Klausen/Chiusa) and Etschtal/Val D´Adige (from Prad am Stilfser Joch/Prato allo Stelvio to Salurn/Salorno) (Fig. 1). Exact locations are provided in Suppl. material 1 and are also part of the monitoring programme for other taxa, including vascular plants, grasshoppers, butterflies, birds, bats and others, conducted by the “Biodiversity Monitoring South Tyrol” (Hilpold et al. 2023).

For bees collected during the first year, a specimen (of both sexes, if present) per species per site was prepared to be stored in an insect box. In the second year, we prepared one specimen (of both sexes, if present) per species per site. All the other bees are labelled, temporarily stored in 70% ethanol and checked regularly in case ethanol refilling was necessary. The specimens will be temporarily kept by the Institute for Alpine Environment at Eurac Research for further research and then gifted to the South Tyrol Museum of Nature in Bolzano/Bozen. Abundance data for each species in each study year are provided in Suppl. material 3; for detailed sampling methodology and habitat descriptions, see Suppl. material 4. The first checklist provides the list of wild bee species, while Suppl. material 2 includes details on their sociality, nesting strategies, dietary preferences and threat status.

### Taxonomic framework

Wild bee specimens were identified at species level using identification keys provided by Amiet (1996), Amiet et al. (1999), Amiet et al. (2001), Amiet et al. (2004), Amiet et al. (2007), Amiet et al. (2010), Bogusch and Straka (2012), Ebmer (1969), Ebmer (1971), Ebmer (1984), Mauss (1994) and Scheuchl (1995), Scheuchl (2006). Females of *Halictussimplex* Blüthgen, 1923 were difficult to differentiate from *H.longobardicus* Blüthgen, 1944 and *H.eurygnathus* Blüthgen, 1931 (Ebmer 1975). We addressed this group of species as the “*Halictussimplex* group”. Similarly, distinguishing *Hylaeusconfusus* Nylander, 1852 from *H.incongruus* Foster 1871 and *H.gibbus* Saunders, 1850 proved challenging (Le Divelec 2022). Therefore, we refer to this group as Hylaeuscf.confusus. Morphological differentiation between *Andrenabarbareae* Panzer, 1805 and the closely-related *Andrenacineraria* (Linnaeus, 1758) was also problematic, so we treat it as Andrenacf.barbareae. Lastly, it is worth mentioning that *Andrenaspinigera* (Kirby, 1802) is considered a distinct species from *A.trimmerana* (Kirby, 1802) by German and Swiss apidologists, though recent barcoding efforts found no genetic differences between the two (Villalta et al. 2021).

## Data resources

The data underpinning the analysis reported in this paper are deposited at GBIF, the Global Biodiversity Information Facility, https://doi.org/10.15468/h4r92a

## Checklists

### Wild bee species list

#### Andrena alfkenella

Perkins, 1914

C8C35231-FC62-53A1-9CA2-BFC5B442D038

##### Notes

2022, Burggrafenamt

#### Andrena barbareae

(cf.) Panzer, 1805

BC074122-1A5F-5C4E-8AAC-272EF3F23EA8

##### Notes

2021 and 2022, Überetsch-Unterland

#### Andrena bicolor

Fabricius, 1775

2435AF3F-BFCB-54A3-80A4-81EF7E7B8A76

##### Notes

2022, Salten-Schlern, Burggrafenamt, Überetsch-Unterland

#### Andrena carantonica

Pérez, 1902

AC6A65A1-7840-5794-9EE7-710260835B22

##### Notes

2021, Vinschgau, Burggrafenamt, Salten-Schlern, Überetsch-Unterland

#### Andrena combinata

(Christ, 1791)

25881AC1-7988-5BE7-94EA-C5964D8823FA

##### Notes

2022, Burggrafenamt

#### Andrena curvungula

Thomson, 1870

570B9EFB-745D-59AC-BF67-F4F7E2BC8B43

##### Notes

2022, Burggrafenamt, Überetsch-Unterland, Salten-Schlern

#### Andrena dorsata

(Kirby, 1802)

5CA18BE7-24E8-54BD-B3E5-2FE569423EEA

##### Notes

2021, Überetsch-Unterland, Vinschgau, Burggrafenamt, Salten-Schlern; 2022, Burggrafenamt, Salten-Schlern

#### Andrena falsifica

Perkins, 1915

76C2D91E-6537-5F7A-ABA2-33ED57AADBEC

##### Notes

2022, Burggrafenamt, Salten-Schlern, Überetsch-Unterland

#### Andrena flavipes

Panzer, 1799

48726C37-E602-5844-A1CB-ACEAA60344D8

##### Notes

2021, Überetsch-Unterland; 2022, Überetsch-Unterland, Salten-Schlern, Burggrafenamt

#### Andrena fulva

(Müller, 1766)

1BEC48F0-207F-5F4C-825F-84528FB0B8D4

##### Notes

2021, Vinschgau, Burggrafenamt, Salten-Schlern, Überetsch-Unterland

#### Andrena fulvago

(Christ, 1791)

B0B66EA9-47CE-50FE-8DB1-6D1DFED843E9

##### Notes

2022, Bozen, Salten-Schlern, Überetsch-Unterland, Burggrafenamt

#### Andrena gelriae

Van der Vecht, 1927

04424E72-E68C-573E-8DD4-EF6E1BFBA900

##### Notes

2022, Burggrafenamt

#### Andrena haemorrhoa

(Fabricius, 1781)

293DE505-720D-5740-A1F9-964049E0D3B6

##### Notes

2021, Überetsch-Unterland, Burggrafenamt, Vinschgau, Salten-Schlern; 2022, Überetsch-Unterland

#### Andrena hattorfiana

(Fabricius, 1775)

408E5A8C-0355-582B-91B3-A6A0962F9927

##### Notes

2022, Burggrafenamt, Überetsch-Unterland, Salten-Schlern

#### Andrena humilis

Imhoff, 1832

F5E33ACD-CFEA-573D-8D2F-FD413812235A

##### Notes

2022, Salten-Schlern, Überetsch-Unterland, Burggrafenamt

#### Andrena labiata

Fabricius, 1781

AF7A6BB7-3B6C-581D-B06B-670EF19BC64A

##### Notes

2022, Burggrafenamt

#### Andrena minutula

(Perkins, 1914)

22DB6B6B-1153-56A0-88DB-6B42425EBE82

##### Notes

2021, Vinschgau, Burggrafenamt, Salten-Schlern, Überetsch-Unterland; 2022, Überetsch-Unterland

#### Andrena minutuloides

(Kirby, 1802)

F94C9DC1-D3D8-5210-9411-778883C06C52

##### Notes

2022, Überetsch-Unterland

#### Andrena nigroaenea

(Kirby, 1802)

08BBD287-A050-5916-B6B5-4DACC153A35B

##### Notes

2021, Überetsch-Unterland, Vinschgau, Burggrafenamt, Salten-Schlern; 2022, Salten-Schlern, Bozen, Burggrafenamt, Überetsch-Unterland

#### Andrena nitida

(Müller, 1776)

A7190D6C-35ED-5CEE-B41C-B5B04BE49A1C

##### Notes

2021, Salten-Schlern

#### Andrena ovatula

(Kirby, 1802) [species group]

BA5ED4E2-D22B-5930-8B12-61BFE3708903

##### Notes

2021, Überetsch-Unterland, Vinschgau; 2022, Überetsch-Unterland

#### Andrena pandellei

Pérez, 1895

1A08ADA6-261B-5DDF-BFA7-97BF9EB4D30C

##### Notes

2022, Burggrafenamt

#### Andrena saxonica

Stöckhert, 1935

94EE25C2-9C93-552D-A506-7046209BBD4F

##### Notes

2021 and 2022, Überetsch-Unterland

#### Andrena schencki

Morawitz, 1866

B0C9FDE8-F860-5EF4-8999-5D7A06BD2FEB

##### Notes

2022, Überetsch-Unterland

#### Andrena simontornyella

Noskiewicz, 1939

02AAB6D2-C333-5FBB-A1C4-1436FDF38DD0

##### Notes

2021, Überetsch-Unterland

#### Andrena spinigera

Kirby, 1802

14CE2682-43E3-52D3-A3FE-109E6BD9CB57

##### Notes

2021 and 2022, Überetsch-Unterland

#### Andrena subopaca

Nylander, 1848

EE82B80C-DB36-5E51-8592-6FAE47D9BA16

##### Notes

2021, Burggrafenamt

#### Andrena symphyti

Schmiedeknecht, 1883

68D75F93-E70E-5D8E-847B-105A27B942F0

##### Notes

2022, Überetsch-Unterland

#### Andrena taraxaci

Giraud, 1861

E364D8E9-4C17-5C01-B685-62367B0097CD

##### Notes

2021, Burggrafenamt, Überetsch-Unterland; 2022, Überetsch-Unterland

#### Andrena thoracica

(Fabricius, 1775)

1FB18549-1BB9-55A7-A215-AD889B38106D

##### Notes

2022, Burggrafenamt

#### Andrena vaga

Panzer, 1799

C6485846-1EC9-56C6-8237-B0295DF1A904

##### Notes

2021, Salten-Schlern

#### Andrena ventralis

Imhoff, 1832

E9263DC5-454A-5D02-8D30-0FDF5DF842FB

##### Notes

2021, Salten-Schlern

#### Andrena wilkella

(Kirby, 1802)

6A5861B8-2D6D-5C42-AF28-1195CF0FE50F

##### Notes

2022, Überetsch-Unterland

#### Anthidium manicatum

(Linné, 1758)

25D4A88E-9588-52D4-B279-E3178AC8AAC3

##### Notes

2022, Burggrafenamt

#### Anthidium nanum

Mocsary, 1881

325F64AE-E1FC-5E24-9162-158195201B96

##### Notes

2022, Burggrafenamt

#### Anthidium oblongatum

(Illiger, 1806)

AD94E29C-0E73-5080-B618-C8E43DAA7B7E

##### Notes

2022, Überetsch-Unterland

#### Anthidium punctatum

Latreille, 1809

6C4619A4-015B-525B-A92C-8C7534D663E3

##### Notes

2022, Salten-Schlern

#### Anthophora plumipes

(Pallas, 1772)

53787F94-DBBF-5C64-9207-FE8262DA0C9D

##### Notes

2021, Burggrafenamt

#### Bombus barbutellus

(Kirby, 1802)

24DBBDF2-2556-5633-95C8-88A8D6B2761A

##### Notes

2022, Burggrafenamt, Salten-Schlern

#### Bombus bohemicus

Seidl, 1838

FBBAC703-9A55-552B-9213-21D5F4E9EAF8

##### Notes

2021, Salten-Schlern; 2022, Burggrafenamt, Salten-Schlern, Überetsch-Unterland

#### Bombus campestris

(Panzer, 1801)

039399B6-FE1F-58D6-9DC4-A1CFAAA5A174

##### Notes

2022, Burggrafenamt

#### Bombus cryptarum

(Fabricius, 1775)

9B1C8E64-A82E-5B61-95BA-C9AE6E722D1E

##### Notes

2022, Salten-Schlern

#### Bombus hortorum

(Linné, 1761)

730BD529-50A4-5CC0-92BF-36CF8B2DB8DA

##### Notes

2022, Burggrafenamt

#### Bombus jonellus

(Kirby, 1802)

ED48B4C7-307E-5D03-8B94-B86ACD350A8A

##### Notes

2022, Überetsch-Unterland

#### Bombus lapidarius

(Linnaeus, 1758)

98B23ED1-94CF-5CE8-A105-6DEB01B78A22

##### Notes

2021, Vinschgau; 2022, Bozen, Überetsch-Unterland, Salten-Schlern, Burggrafenamt

#### Bombus lucorum

(Linnaeus, 1761)

513D2080-2BE3-527E-9F3A-ACA4E4072B85

##### Notes

2021, Vinschgau, Salten-Schlern, Burggrafenamt; 2022, Bozen, Überetsch-Unterland, Salten-Schlern, Burggrafenamt

#### Bombus pascuorum

(Scopoli, 1763)

0046F888-1050-53AD-BF6F-8080BEBB25E2

##### Notes

2022, Bozen, Überetsch-Unterland, Salten-Schlern, Burggrafenamt

#### Bombus pratorum

(Linnaeus, 1761)

86B60C68-F7E9-5F14-B099-98C4AC43CCD1

##### Notes

2021, Salten-Schlern; 2022, Überetsch-Unterland, Salten-Schlern, Burggrafenamt

#### Bombus soroeensis

(Fabricius, 1776)

046C161E-69E8-5737-8654-011D3EF2AE1B

##### Notes

2022, Überetsch-Unterland, Burggrafenamt

#### Bombus sylvestris

(Lepeletier, 1832)

7B861D1E-1F9B-5666-9964-678604A43067

##### Notes

2021, Salten-Schlern; 2022, Salten-Schlern, Burggrafenamt

#### Bombus terrestris

(Linnaeus, 1758)

B505FFD4-0A9B-5B42-9949-6D2AE581F839

##### Notes

2021, Vinschgau; 2022, Bozen, Überetsch-Unterland, Salten-Schlern, Burggrafenamt

#### Ceratina chalybea

Chevrier, 1872

9A8D9852-7551-53AA-8120-AADC8324178B

##### Notes

2022, Überetsch-Unterland, Salten-Schlern, Burggrafenamt

#### Ceratina cucurbitina

(Rossi, 1792)

E7FC5915-4BCA-5B79-8AC3-204D5A1BE435

##### Notes

2021, Burggrafenamt; 2022, Bozen, Überetsch-Unterland, Salten-Schlern, Burggrafenamt

#### Ceratina cyanea

(Kirby, 1802)

E35F4324-D3D9-5E97-8891-0110DD59A196

##### Notes

2021, Salten-Schlern; 2022, Überetsch-Unterland, Salten-Schlern, Burggrafenamt

#### Chelostoma distinctum

(Stöckhert, 1929)

6610EA9B-A88D-533B-8BAA-FA54855C5D01

##### Notes

2022, Bozen, Überetsch-Unterland, Salten-Schlern, Burggrafenamt

#### Chelostoma florisomne

(Linnaeus, 1758)

8D45927A-BC4B-5692-A03C-614D6A0CB2B5

##### Notes

2021, Burggrafenamt; 2022, Überetsch-Unterland, Burggrafenamt

#### Chelostoma foveolatum

(Morawitz, 1868)

D94075FC-9B42-5A2E-8795-A88C760E4098

##### Notes

2022, Burggrafenamt

#### Chelostoma rapunculi

(Lepeletier, 1841)

9C56CDC1-8445-55E1-84E1-CD71036026B7

##### Notes

2022, Salten-Schlern, Burggrafenamt

#### Dufourea dentiventris

(Nylander, 1848)

B3B275C8-DCD7-5CE7-AED0-4FA8E30DDF9F

##### Notes

2022, Salten-Schlern, Burggrafenamt

#### Dufourea inermis

(Nylander, 1848)

6EEBC31C-4C40-5E3E-9315-22DE67C9562B

##### Notes

2022, Burggrafenamt

#### Eucera longicornis

(Linné, 1758)

94A5BE15-5673-59E5-8B42-605B9147C4E3

##### Notes

2022, Überetsch-Unterland, Salten-Schlern

#### Eucera nigrescens

Pérez, 1879

69CFB1D7-9DFB-54F5-8B67-392839013656

##### Notes

2021, Vinschgau, Überetsch-Unterland; 2022, Überetsch-Unterland, Burggrafenamt

#### Halictus langobardicus

Blüthgen, 1944

CB165BAD-1F0E-51A1-8966-ED1C367D67A7

##### Notes

2022, Überetsch-Unterland

#### Halictus maculatus

Smith, 1848

5C3927C1-5193-5A73-BA83-B84F2324518D

##### Notes

2021, Vinschgau; 2022, Überetsch-Unterland, Salten-Schlern, Burggrafenamt

#### Halictus rubicundus

(Christ, 1791)

AE776916-E4DF-5C92-AC35-BB0A51549A10

##### Notes

2022, Burggrafenamt

#### Halictus sexcinctus

(Fabricius, 1775)

834B1675-FA6C-55C0-AF61-60E85080709D

##### Notes

2022, Bozen, Überetsch-Unterland, Salten-Schlern, Burggrafenamt

#### Halictus simplex

(cf.) Blüthgen, 1923

5AE1D23A-4E21-579D-91DF-723E554501F5

##### Notes

2021, Vinschgau; 2022, Bozen, Überetsch-Unterland, Salten-Schlern, Burggrafenamt

#### Halictus subauratus

(Rossi, 1792)

7CB800DE-8E66-5199-9CFA-A865F8F39EFC

##### Notes

2022, Bozen, Überetsch-Unterland, Salten-Schlern, Burggrafenamt

#### Halictus tumulorum

(Linné, 1758)

8C70938C-AE3E-5A42-8D79-EE758D1E1FB7

##### Notes

2022, Überetsch-Unterland, Salten-Schlern, Burggrafenamt

#### Heriades truncorum

(Linnaeus, 1758)

84D71E5D-517F-5FE8-A96D-890F30B2C040

##### Notes

2022, Überetsch-Unterland, Salten-Schlern, Burggrafenamt

#### Hylaeus angustatus

(Schenck, 1861)

2482CDED-B8EB-5284-9EA3-20BBE5D758C8

##### Notes

2022, Bozen, Salten-Schlern, Burggrafenamt

#### Hylaeus brevicornis

Nylander, 1852

0B9A3760-3DC5-504F-983B-DC155D7D6F7A

##### Notes

2021, Überetsch-Unterland; 2022, Überetsch-Unterland, Salten-Schlern, Burggrafenamt

#### Hylaeus communis

Nylander, 1852

397B48B7-9CF4-596D-827C-CBAD736A056B

##### Notes

2022, Überetsch-Unterland, Salten-Schlern, Burggrafenamt

#### Hylaeus confusus

(cf.) Nylander, 1852

92178D65-BFF3-5E06-B3C6-717A4325E503

##### Notes

2021, Vinschgau, Überetsch-Unterland, Burggrafenamt; 2022, Bozen, Überetsch-Unterland, Salten-Schlern, Burggrafenamt

#### Hylaeus difformis

(Eversmann, 1852)

26B409FC-A506-5EDF-A215-9BC97E577B9A

##### Notes

2022, Salten-Schlern

#### Hylaeus dilatatus

(Kirby, 1802)

52EE1BA3-1F13-5FD1-B271-E290E8547558

##### Notes

2022, Salten-Schlern, Burggrafenamt

#### Hylaeus gredleri

Förster, 1871

64676598-B685-505F-9BAB-B1506656B039

##### Notes

2022, Burggrafenamt

#### Hylaeus hyalinatus

Smith, 1842

FACDCD9D-CE37-56F4-8EC7-15EE37450456

##### Notes

2022, Salten-Schlern, Burggrafenamt

#### Hylaeus kahri

Förster, 1871

87BE817A-D712-5F1B-8AF1-67177274CCAA

##### Notes

2022, Salten-Schlern, Burggrafenamt

#### Hylaeus leptocephalus

(Morawitz, 1870)

F8E4856D-C16F-531B-BC95-E5CDA6B1D3D2

##### Notes

2022, Überetsch-Unterland

#### Hylaeus nigritus

(Fabricius, 1798)

0B208054-8FD0-5A03-BE74-FE8112050FD0

##### Notes

2022, Salten-Schlern

#### Hylaeus sinuatus

(Schenck, 1853)

2693054C-D253-5F26-BA72-E69BD1284EE2

##### Notes

2022, Salten-Schlern, Burggrafenamt

#### Hylaeus styriacus

Förster, 1871

F913C617-CC56-5960-8AD4-D72F91A312AA

##### Notes

2022, Salten-Schlern

#### Hylaeus variegatus

(Fabricius, 1798)

E69347D5-B263-56C5-AB2D-6042643C23A9

##### Notes

2022, Salten-Schlern, Bozen

#### Lasioglossum aeratum

(Kirby, 1802)

11051003-E78E-5A19-8EC3-82D3E1936FE0

##### Notes

2022, Salten-Schlern, Burggrafenamt

#### Lasioglossum albipes

(Fabricius, 1781)

E2D84390-7347-5617-BFFB-725B291E4D63

##### Notes

2022, Burggrafenamt, Salten-Schlern, Überetsch-Unterland

#### Lasioglossum bluethgeni

Ebmer, 1971

8E324FB8-E87E-5FCD-9242-0F503DE7EA9A

##### Notes

2022, Burggrafenamt, Salten-Schlern, Überetsch-Unterland

#### Lasioglossum brevicorne

(Schenck, 1870)

C335C52E-2BC4-5300-9CF9-8FE452866826

##### Notes

2022, Burggrafenamt, Salten-Schlern

#### Lasioglossum calceatum

(Scopoli, 1763)

94A784A5-4F1B-509D-AAB3-F026AD53D492

##### Notes

2021, Vinschgau, Burggrafenamt, Salten-Schlern, Überetsch-Unterland; 2022, Burggrafenamt, Salten-Schlern, Überetsch-Unterland

#### Lasioglossum fulvicorne

(Kirby, 1802)

DB434632-6589-5B72-84F7-FF69EEAF05F1

##### Notes

2021, Vinschgau, Salten-Schlern, Überetsch-Unterland; 2022, Burggrafenamt, Salten-Schlern, Überetsch-Unterland

#### Lasioglossum glabriusculum

(Morawitz, 1872)

B0A2079F-280A-503A-B494-18E4EE9C74E5

##### Notes

2021, Überetsch-Unterland; 2022, Salten-Schlern, Überetsch-Unterland

#### Lasioglossum laevigatum

(Kirby, 1802)

285ED4C4-F9B2-557B-89D1-9A81D9CFC574

##### Notes

2022, Burggrafenamt, Salten-Schlern, Überetsch-Unterland

#### Lasioglossum laterale

(Brullé, 1832)

D1F04ABD-2B6A-5827-9FE2-4D983A659D20

##### Notes

2021 and 2022, Überetsch-Unterland

#### Lasioglossum laticeps

(Schenck, 1870)

BF0075DB-F96F-5EE8-872A-26CAF491146A

##### Notes

2021, Vinschgau, Burggrafenamt, Salten-Schlern, Überetsch-Unterland; 2022, Burggrafenamt, Salten-Schlern, Überetsch-Unterland

#### Lasioglossum lativentre

(Schenck, 1853)

C5B9324E-363B-553B-B4F8-8EAA426654F6

##### Notes

2021, Salten-Schlern; 2022, Burggrafenamt, Salten-Schlern

#### Lasioglossum leucopus

(Kirby, 1802)

BB53F67C-615E-5BA9-8D8A-815A62875C73

##### Notes

2022, Burggrafenamt, Salten-Schlern, Überetsch-Unterland

#### Lasioglossum leucozonium

(Schrank, 1781)

19A59386-CE8B-54AC-AEDD-75AC8AC5E918

##### Notes

2022, Bozen, Burggrafenamt, Salten-Schlern, Überetsch-Unterland

#### Lasioglossum lissonotum

(Noskiewicz, 1926)

E085AEDC-7C9A-5CF0-8DE5-516342C0B3B0

##### Notes

2022, Burggrafenamt

#### Lasioglossum minutissimum

(Kirby, 1802)

DEFB4C04-40B5-52B3-9903-9D413A5C3803

##### Notes

2021 and 2022, Burggrafenamt, Überetsch-Unterland

#### Lasioglossum monstrificum

(Morawitz, 1891)

9FEA714D-8249-52D3-B9B8-BCAD3D8AFBC4

Syn. *L.sabulosum* (Warncke, 1986)

##### Notes

2021, Salten-Schlern

#### Lasioglossum morio

(Fabricius, 1793)

782DBF95-5E74-5305-A6F6-7F3DDC3F4027

##### Notes

2021, Vinschgau, Burggrafenamt, Salten-Schlern, Überetsch-Unterland; 2022, Bozen, Burggrafenamt, Salten-Schlern, Überetsch-Unterland

#### Lasioglossum nitidulum

(Fabricius, 1804)

93AAF2AA-1F7C-54F1-90DD-42DBAB69549E

##### Notes

2022, Bozen, Burggrafenamt, Salten-Schlern, Überetsch-Unterland

#### Lasioglossum pauxillum

(Schenck, 1853)

4A13A431-CD08-5377-A005-FC3A953117F9

##### Notes

2021, Burggrafenamt, Überetsch-Unterland; 2022, Bozen, Burggrafenamt, Salten-Schlern, Überetsch-Unterland

#### Lasioglossum politum

(Schenck, 1853)

3FEAE5A4-AB84-5777-9CBD-E839F0806D37

##### Notes

2021, Überetsch-Unterland; 2022, Salten-Schlern

#### Lasioglossum punctatissimum

(Schenck, 1853)

C9FC1781-8712-5CAE-BE7C-843052F1CAC9

##### Notes

2021, Burggrafenamt, Überetsch-Unterland; 2022, Burggrafenamt, Salten-Schlern, Überetsch-Unterland

#### Lasioglossum rufitarse

(Zetterstedt, 1838)

8BC3CB96-5A59-50B3-9552-570512C1ED01

##### Notes

2022, Burggrafenamt

#### Lasioglossum transitorium

(Schenck, 1868)

BC514E06-6981-5E98-8D6C-AF95CD9D25B9

##### Notes

2021, Vinschgau, Überetsch-Unterlan; 2022, Bozen, Salten-Schlern

#### Lasioglossum villosulum

(Kirby, 1802)

CD2DA482-58BA-56BE-B456-C7DB695E70B9

##### Notes

2022, Burggrafenamt, Salten-Schlern, Überetsch-Unterland

#### Lasioglossum zonulum

(Smith, 1848)

565F8E54-06EA-5C33-A2B0-26327DF1416E

##### Notes

2021, Burggrafenamt, Vinschgau, Überetsch-Unterland; 2022, Burggrafenamt, Salten-Schlern, Überetsch-Unterland

#### Megachile centuncularis

(Linné, 1758)

7DD78612-6C57-5785-ADAD-FD95B7FA385C

##### Notes

2022, Burggrafenamt, Überetsch-Unterland

#### Megachile melanopyga

Costa, 1863

83FF8554-EE9F-52F8-A692-D07E43C5B760

##### Notes

2022, Überetsch-Unterland

#### Megachile parietina

(Geoffroy, 1785)

62CF6A54-B796-5281-8CC7-11075755E28E

##### Notes

2022, Salten-Schlern

#### Megachile pilidens

Alfken, 1924

D57B68F9-D53E-5229-816F-DCC0038CF062

##### Notes

2022, Burggrafenamt, Bozen

#### Megachile rotundata

(Fabricius, 1787)

3D922041-1056-5DD3-94BF-47631F101BDB

##### Notes

2022, Burggrafenamt

#### Megachile versicolor

Smith, 1844

525DDB4A-9AE8-544A-A6D3-EDDE361ECA67

##### Notes

2021, Überetsch-Unterland; 2022, Burggrafenamt

#### Megachile willughbiella

(Kirby,1802)

A3A56328-DD8D-5206-AFC4-3AE85D44D171

##### Notes

2021, Vinschgau

#### Melecta albifrons

(Forster, 1771)

E3FE104E-708A-598C-8933-CCE491496224

##### Notes

2022, Überetsch-Unterland

#### Melecta luctuosa

(Scopoli, 1770)

71ABB490-4679-5259-A7E7-6F0B9FEFFD72

##### Notes

2022, Bozen

#### Melitta haemorrhoidalis

(Fabricius, 1775)

7D9AAE49-23EE-5BC0-A550-5C8252BE45BA

##### Notes

2022, Burggrafenamt, Salten-Schlern

#### Melitta leporina

(Panzer, 1799)

ECB26C5F-58FE-596C-A43A-BA2FC1354211

##### Notes

2022, Burggrafenamt, Salten-Schlern

#### Nomada emarginata

Morawitz, 1877

18954C53-755F-50BC-85E2-315EC073C236

##### Notes

2022, Burggrafenamt

#### Nomada flava

Panzer, 1798

E707A3D9-017A-5AD8-BD74-423993C0E44C

##### Notes

2021, Burggrafenamt

#### Nomada flavoguttata

(Kirby, 1802)

63551B34-749C-5B80-AFC2-05D32FC99C4F

##### Notes

2022, Überetsch-Unterland

#### Nomada flavopicta

(Kirby, 1802)

DD8E8757-5681-5111-886B-95F18E4855EF

##### Notes

2022, Burggrafenamt, Salten-Schlern

#### Nomada integra

Brullé, 1832

105803C2-C664-5196-B56F-9BC0534ED782

##### Notes

2022, Salten-Schlern

#### Nomada mutica

Morawitz, 1872

AF071051-2187-552A-AA04-339FEE490C1F

##### Notes

2021, Vinschgau

#### Nomada sexfasciata

Panzer, 1799

8644B4E6-45CD-5538-8B96-110825A8FE8D

##### Notes

2022, Salten-Schlern, Überetsch-Unterland

#### Nomada signata

Jurine 1807

37C1BE18-2579-59BC-9AFD-B8FC40F9B989

##### Notes

2021, Burggrafenam

#### Nomada villosa

Thomson, 1870

313E8280-16C4-5F12-A764-1C25B3ECF75B

##### Notes

2022, Salten-Schlern

#### Nomada zonata

Panzer 1798

CED6ED99-02C9-5D15-BEDA-3066CF5BCACB

##### Notes

2021, Burggrafenam

#### Osmia bicornis

(Linnaeus, 1758)

421AA3BB-CAA9-5CEB-86F2-EE38FB86975A

##### Notes

2021, Überetsch-Unterland, Burggrafenamt, Vinschgau; 2022, Bozen, Überetsch-Unterland, Burggrafenamt, Salten-Schlern

#### Osmia brevicornis

(Fabricius, 1798)

33874EAA-4501-5BE6-A0A1-E48D0949262F

##### Notes

2022, Burggrafenamt

#### Osmia caerulescens

(Linné, 1758)

DC5ECFAC-7804-5ED4-AA7F-E241A20CDEF3

##### Notes

2022, Überetsch-Unterland, Burggrafenamt

#### Osmia cornuta

(Latreille, 1805)

73E60022-7982-54A4-A241-8F6D5AA4C0E7

##### Notes

2021, Burggrafenamt

#### Osmia gallarum

Spinola, 1808

3D548A6E-8DAA-59AB-83C0-39E2108EF301

##### Notes

2022, Überetsch-Unterland, Burggrafenamt

#### Osmiav leucomelana

(Kirby, 1802)

4307B03F-9169-5A4F-A5EF-0637806BBD5A

##### Notes

2022, Überetsch-Unterland, Burggrafenamt

#### Osmia mitis

Nylander, 1852

8A644884-9EBF-5C78-9ACB-7C9C5268F4E0

##### Notes

2022, Überetsch-Unterland, Burggrafenamt

#### Osmia mustelina

Gerstaecker, 1869

51DC22D8-B4E9-5422-B6B0-D685D1FE9A21

##### Notes

2022, Salten-Schlern

#### Osmia rufohirta

Latreille, 1811

B7014731-248B-5AFF-811B-24EA12C9B3DC

##### Notes

2022, Bozen, Burggrafenamt

#### Osmia tridentata

Dufour & Perris, 1840

FA728FBC-5D97-59B4-B934-DDC37BD24B84

##### Notes

2022, Überetsch-Unterland

#### Osmia tuberculata

Nylander, 1848

C7FE41FC-0DF6-54B4-8F94-48A2CDEBD422

##### Notes

2022, Salten-Schlern

#### Panurgus banksianus

(Kirby, 1802)

83DE30F9-23A1-5C3A-B89A-582274290546

##### Notes

2022, Überetsch-Unterland, Burggrafenamt, Salten-Schlern

#### Panurgus calcaratus

(Scopoli, 1763)

604D6635-20E7-57F8-827F-AA6206E3DF4D

##### Notes

2022, Überetsch-Unterland, Salten-Schlern

#### Sphecodes ephippius

(Linnaeus, 1767)

BA2050C6-1D84-553D-8B76-0A6A9A806AE4

##### Notes

2021, Vinschgau, Burggrafenamt; 2022, Burggrafenamt

#### Sphecodes ferruginatus

Hagens, 1882

920D324B-B518-5238-8F97-40A42DDB89EA

##### Notes

2022, Burggrafenamt

#### Sphecodes monilicornis

(Kirby, 1802)

D1ABDC34-3E3A-51CD-B7A1-5B90B3FA9E1C

##### Notes

2022, Überetsch-Unterland

#### Sphecodes niger

Hagens, 1874

3A54D619-0753-5244-A558-60E92F88B35C

##### Notes

2022, Burggrafenamt

#### Stelis ornatula

(Klug, 1807)

548DF887-FBAF-520D-925E-DD36A95A6BE0

##### Notes

2022, Burggrafenamt

#### Xylocopa valga

Gerstaecker, 1872

48D495B2-C4C8-5E6B-BDEA-46A9AC7F5F80

##### Notes

2022, Überetsch-Unterland

#### Xylocopa violacea

(Linné, 1758)

46064AD7-98F4-5E95-8168-F17246590BB9

##### Notes

2022, Burggrafenamt

### Near threatened species according to the European Red List of Bees

#### Andrena hattorfiana

(Fabricius, 1775)

57239AE3-89C1-5CC1-B795-A8E78D3B3D8F

##### Materials

- **Type status:** Other material. **Occurrence:** occurrenceRemarks: blue pan trap; recordedBy: S. Zanini; individualCount: 1; sex: male; occurrenceID: 0846AC6F-A292-5A4B-9BFE-5A1CE0EDFAE3; **Location:** country: Italy; stateProvince: South Tyrol; verbatimLocality: Tisens; verbatimElevation: 1430 m; decimalLatitude: 46.547390; decimalLongitude: 11.115870; **Identification:** identifiedBy: T. Kopf; **Event:** samplingProtocol: pan trap; eventDate: 14/6/2022; habitat: hay meadow
- **Type status:** Other material. **Occurrence:** occurrenceRemarks: blue pan trap; recordedBy: S. Zanini; individualCount: 1; sex: female; occurrenceID: FC544941-6974-5DC8-96CF-56D4C9318AA8; **Location:** country: Italy; stateProvince: South Tyrol; verbatimLocality: Gfrill; verbatimElevation: 1267 m; decimalLatitude: 46.275860; decimalLongitude: 11.282120; **Identification:** identifiedBy: T. Kopf; **Event:** samplingProtocol: pan trap; eventDate: 12/6/2022; habitat: hay meadow
- **Type status:** Other material. **Occurrence:** occurrenceRemarks: blue pan trap; recordedBy: S. Zanini; individualCount: 1; sex: female; occurrenceID: A2A27312-BDD6-5634-A685-A00E0972807A; **Location:** country: Italy; stateProvince: South Tyrol; verbatimLocality: Sinich; verbatimElevation: 276 m; decimalLatitude: 46.640990; decimalLongitude: 11.153260; **Identification:** identifiedBy: T. Kopf; **Event:** samplingProtocol: pan trap; eventDate: 10/7/2022; habitat: apple orchard
- **Type status:** Other material. **Occurrence:** occurrenceRemarks: blue pan trap; recordedBy: S. Zanini; individualCount: 3; sex: 3 females; occurrenceID: 7C27E016-61CA-536E-971A-CA8CD37B35EE; **Location:** country: Italy; stateProvince: South Tyrol; verbatimLocality: Unterinn; verbatimElevation: 901 m; decimalLatitude: 46.506610; decimalLongitude: 11.424850; **Identification:** identifiedBy: T. Kopf; **Event:** samplingProtocol: pan trap; eventDate: 13/7/2022; habitat: orchard meadow
- **Type status:** Other material. **Occurrence:** occurrenceRemarks: blue pan trap; recordedBy: S. Zanini; individualCount: 4; sex: 4 females; occurrenceID: 64F8427E-1AF7-5751-9F20-BA640E69F54B; **Location:** country: Italy; stateProvince: South Tyrol; verbatimLocality: Siffian; verbatimElevation: 1055 m; decimalLatitude: 46.535360; decimalLongitude: 11.473600; **Identification:** identifiedBy: T. Kopf; **Event:** samplingProtocol: pan trap; eventDate: 13/7/2022; habitat: crop field
- **Type status:** Other material. **Occurrence:** occurrenceRemarks: blue pan trap; recordedBy: S. Zanini; individualCount: 1; sex: male; occurrenceID: 174051B0-D2E5-58BB-9742-02046497EF24; **Location:** country: Italy; stateProvince: South Tyrol; verbatimLocality: Salurn; verbatimElevation: 209 m; decimalLatitude: 46.251770; decimalLongitude: 11.204790; **Identification:** identifiedBy: T. Kopf; **Event:** samplingProtocol: pan trap; eventDate: 8/7/2022; habitat: vineyard
- **Type status:** Other material. **Occurrence:** occurrenceRemarks: blue pan trap; recordedBy: S. Zanini; individualCount: 1; sex: female; occurrenceID: EDA31884-143E-5475-A72B-5F9DAD9AB365; **Location:** country: Italy; stateProvince: South Tyrol; verbatimLocality: Prissian; verbatimElevation: 770 m; decimalLatitude: 46.551600; decimalLongitude: 11.168630; **Identification:** identifiedBy: T. Kopf; **Event:** samplingProtocol: pan trap; eventDate: 14/6/2022; habitat: orchard meadow

##### Notes

solitary behaviour, nests below-ground, oligolectic (Caprifoliaceae).

#### Dufourea dentiventris

(Nylander, 1848)

2E969B58-0976-5715-B5EC-1EFDE7B90616

##### Materials

- **Type status:** Other material. **Occurrence:** occurrenceRemarks: blue pan trap; recordedBy: S. Zanini; individualCount: 1; sex: male; occurrenceID: 6F1E8C93-A927-57FB-80E6-6F8DF94E163E; **Location:** country: Italy; stateProvince: South Tyrol; verbatimLocality: Deutschnofen; verbatimElevation: 1333 m; decimalLatitude: 46.419560; decimalLongitude: 11.501210; **Identification:** identifiedBy: T. Kopf; **Event:** samplingProtocol: pan trap; eventDate: 12/6/2022; habitat: hay meadow
- **Type status:** Other material. **Occurrence:** occurrenceRemarks: blue pan trap; recordedBy: S. Zanini; individualCount: 1; sex: female; occurrenceID: A6795E44-78C2-5E59-A42A-93D26C4CC07A; **Location:** country: Italy; stateProvince: South Tyrol; verbatimLocality: Deutschnofen; verbatimElevation: 1333 m; decimalLatitude: 46.419560; decimalLongitude: 11.501210; **Identification:** identifiedBy: T. Kopf; **Event:** samplingProtocol: pan trap; eventDate: 12/6/2022; habitat: hay meadow
- **Type status:** Other material. **Occurrence:** occurrenceRemarks: yellow pan trap; recordedBy: S. Zanini; individualCount: 1; sex: female; occurrenceID: 80DF03D8-7A94-54A5-AECD-400CD1CE6C1B; **Location:** country: Italy; stateProvince: South Tyrol; verbatimLocality: Tisens; verbatimElevation: 1430 m; decimalLatitude: 46.547390; decimalLongitude: 11.115870; **Identification:** identifiedBy: T. Kopf; **Event:** samplingProtocol: pan trap; eventDate: 17/7/2022; habitat: hay meadow

##### Notes

solitary behaviour, nests below-ground, oligolectic (*Campanula* sp.).

#### Dufourea inermis

(Nylander, 1848)

1C507397-A242-55DA-A4BC-4501A109819D

##### Materials

- **Type status:** Other material. **Occurrence:** occurrenceRemarks: blue pan trap; recordedBy: S. Zanini; individualCount: 1; sex: female; occurrenceID: 81DFB6D9-BCF6-53FA-BC38-32376CB2A162; **Location:** country: Italy; stateProvince: South Tyrol; verbatimLocality: Gargazon; verbatimElevation: 323 m; decimalLatitude: 46.575370; decimalLongitude: 11.217770; **Identification:** identifiedBy: T. Kopf; **Event:** samplingProtocol: pan trap; eventDate: 10/7/2022; habitat: pasture
- **Type status:** Other material. **Occurrence:** occurrenceRemarks: blue pan trap; recordedBy: S. Zanini; individualCount: 1; sex: male; occurrenceID: CF41AD34-A50B-5BFA-928C-6865178199AB; **Location:** country: Italy; stateProvince: South Tyrol; verbatimLocality: Prissian; verbatimElevation: 770 m; decimalLatitude: 46.551600; decimalLongitude: 11.168630; **Identification:** identifiedBy: T. Kopf; **Event:** samplingProtocol: pan trap; eventDate: 15/5/2022; habitat: orchard meadow

##### Notes

solitary behaviour, nests below-ground, oligolectic (*Campanula* sp.).

#### Lasioglossum brevicorne

(Schenck, 1870)

DF6D3BD3-8657-5A92-9B34-3B422E4620A0

##### Materials

- **Type status:** Other material. **Occurrence:** occurrenceRemarks: white pan trap; recordedBy: S. Zanini; individualCount: 1; sex: female; occurrenceID: A8E8BFA2-C655-5B67-A5DE-86748A6B7A98; **Location:** country: Italy; stateProvince: South Tyrol; verbatimLocality: Siffian; verbatimElevation: 1055 m; decimalLatitude: 46.535360; decimalLongitude: 11.473600; **Identification:** identifiedBy: T. Kopf; **Event:** samplingProtocol: pan trap; eventDate: 18/5/2022; habitat: crop field
- **Type status:** Other material. **Occurrence:** occurrenceRemarks: yellow pan trap; recordedBy: S. Zanini; individualCount: 1; sex: female; occurrenceID: 49C71A37-38F7-5131-A658-A5B35FA253F7; **Location:** country: Italy; stateProvince: South Tyrol; verbatimLocality: Siffian; verbatimElevation: 1055 m; decimalLatitude: 46.535360; decimalLongitude: 11.473600; **Identification:** identifiedBy: T. Kopf; **Event:** samplingProtocol: pan trap; eventDate: 13/7/2022; habitat: crop field
- **Type status:** Other material. **Occurrence:** occurrenceRemarks: white pan trap; recordedBy: S. Zanini; individualCount: 3; sex: 3 females; occurrenceID: 1C860E6F-13ED-5902-9BD0-8B802D622BAC; **Location:** country: Italy; stateProvince: South Tyrol; verbatimLocality: Ried; verbatimElevation: 1087 m; decimalLatitude: 46.733800; decimalLongitude: 11.182640; **Identification:** identifiedBy: T. Kopf; **Event:** samplingProtocol: pan trap; eventDate: 22/5/2022; habitat: pasture

##### Notes

sociality unkown (probably solitary), nests below-ground, polylectic.

#### Lasioglossum laevigatum

(Kirby, 1802)

EAB3BA05-85C0-5FEC-81D3-6ABE8C73A4D3

##### Materials

- **Type status:** Other material. **Occurrence:** occurrenceRemarks: white pan trap; recordedBy: S. Zanini; individualCount: 1; sex: female; occurrenceID: 506F84C5-A743-5D07-BD30-FE7145948ADD; **Location:** country: Italy; stateProvince: South Tyrol; verbatimLocality: Tisens; verbatimElevation: 1430 m; decimalLatitude: 46.547390; decimalLongitude: 11.115870; **Identification:** identifiedBy: T. Kopf; **Event:** samplingProtocol: pan trap; eventDate: 14/6/2022; habitat: hay meadow
- **Type status:** Other material. **Occurrence:** occurrenceRemarks: yellow pan trap; recordedBy: S. Zanini; individualCount: 1; sex: female; occurrenceID: 97872A31-3F28-5A8F-A3A5-8E17AA2CB489; **Location:** country: Italy; stateProvince: South Tyrol; verbatimLocality: Tisens; verbatimElevation: 1430 m; decimalLatitude: 46.547390; decimalLongitude: 11.115870; **Identification:** identifiedBy: T. Kopf; **Event:** samplingProtocol: pan trap; eventDate: 14/6/2022; habitat: hay meadow
- **Type status:** Other material. **Occurrence:** occurrenceRemarks: blue pan trap; recordedBy: S. Zanini; individualCount: 1; sex: female; occurrenceID: 81B63674-F5FE-5214-BAE1-083A88D8BC5D; **Location:** country: Italy; stateProvince: South Tyrol; verbatimLocality: Kaltenbrunn; verbatimElevation: 928 m; decimalLatitude: 46.338600; decimalLongitude: 11.357090; **Identification:** identifiedBy: T. Kopf; **Event:** samplingProtocol: pan trap; eventDate: 13/7/2022; habitat: hay meadow
- **Type status:** Other material. **Occurrence:** occurrenceRemarks: white pan trap; recordedBy: S. Zanini; individualCount: 1; sex: female; occurrenceID: EA006B4C-3974-551F-AE14-60938417B51D; **Location:** country: Italy; stateProvince: South Tyrol; verbatimLocality: Siffian; verbatimElevation: 1055 m; decimalLatitude: 46.535360; decimalLongitude: 11.473600; **Identification:** identifiedBy: T. Kopf; **Event:** samplingProtocol: pan trap; eventDate: 18/5/2022; habitat: crop field
- **Type status:** Other material. **Occurrence:** occurrenceRemarks: yellow pan trap; recordedBy: S. Zanini; individualCount: 1; sex: female; occurrenceID: 50C1C572-A98F-5610-89BF-FDA0A382679C; **Location:** country: Italy; stateProvince: South Tyrol; verbatimLocality: Tisens; verbatimElevation: 903 m; decimalLatitude: 46.550230; decimalLongitude: 11.155200; **Identification:** identifiedBy: T. Kopf; **Event:** samplingProtocol: pan trap; eventDate: 20/5/2022; habitat: crop field
- **Type status:** Other material. **Occurrence:** occurrenceRemarks: yellow pan trap; recordedBy: S. Zanini; individualCount: 1; sex: female; occurrenceID: 8C344DC6-7BE8-52C9-AEFB-9443988EC332; **Location:** country: Italy; stateProvince: South Tyrol; verbatimLocality: Tisens; verbatimElevation: 903 m; decimalLatitude: 46.550230; decimalLongitude: 11.155200; **Identification:** identifiedBy: T. Kopf; **Event:** samplingProtocol: pan trap; eventDate: 14/6/2022; habitat: crop field

##### Notes

solitary behaviour, nests below-ground, polylectic.

#### Lasioglossum monstrificum

(Morawitz, 1891)

DBCF2A0D-1A21-52BA-B07F-C284E67F6A88

##### Materials

- **Type status:** Other material. **Occurrence:** occurrenceRemarks: white pan trap; recordedBy: S. Zanini; individualCount: 3; sex: 3 females; occurrenceID: FACD76BC-0926-5505-95D6-2E17E080A57E; **Location:** country: Italy; stateProvince: South Tyrol; verbatimLocality: Albeins; verbatimElevation: 546 m; decimalLatitude: 46.684385; decimalLongitude: 11.638950; **Identification:** identifiedBy: T. Kopf; **Event:** samplingProtocol: pan trap; eventDate: 19/5/2021; habitat: apple orchard

##### Notes

primitively eusocial behaviour, nesting unknown, polylectic.

#### Nomada mutica

Morawitz, 1872

B256A445-7B32-5D67-B270-D3736B110E37

##### Materials

- **Type status:** Other material. **Occurrence:** occurrenceRemarks: white pan trap; recordedBy: S. Zanini; individualCount: 1; sex: male; occurrenceID: 1BABFE2E-5D65-536F-9107-06C137A0AE78; **Location:** country: Italy; stateProvince: South Tyrol; verbatimLocality: Schlanders; verbatimElevation: 694 m; decimalLatitude: 46.618851; decimalLongitude: 10.781159; **Identification:** identifiedBy: T. Kopf; **Event:** samplingProtocol: pan trap; eventDate: 19/5/2021; habitat: apple orchard

##### Notes

parasitic, *Andrenaferox* is the main host bee.

#### Nomada villosa

Thomson, 1870

1C423429-0AEB-5BEB-8430-FCA9504D2266

##### Materials

- **Type status:** Other material. **Occurrence:** occurrenceRemarks: yellow pan trap; recordedBy: S. Zanini; individualCount: 1; sex: female; occurrenceID: 8B0E29C6-0735-5EDE-BBE1-0AA46A7F4F63; **Location:** country: Italy; stateProvince: South Tyrol; verbatimLocality: Unterinn; verbatimElevation: 901 m; decimalLatitude: 46.506610; decimalLongitude: 11.424850; **Identification:** identifiedBy: T. Kopf; **Event:** samplingProtocol: pan trap; eventDate: 18/5/2022; habitat: orchard meadow

##### Notes

parasitic, *Andrenalathyri* is the main host bee.

## Analysis

Overall, we collected 3,313 wild bee specimens across various habitats in South Tyrol, including apple orchards, vineyards, meadows, pastures and arable land (habitat description in Suppl. material 4). We reported 150 wild bee species from 21 genera, sex differentiation and their abundance in the surveyed locations (Suppl. material 3). Additionally, our species list in Suppl. material 2 provides insights into sociality, nesting strategies, and dietary preferences sourced from relevant literature (Scheuchl and Willner 2016, Westrich 2019, Geppert et al. 2023). In the first study, we sampled 792 wild bees representing 55 species, while in the second study, we sampled 2,521 individuals encompassing 135 species. Amongst the sampled species, only 21 out of the 150 were parasitic. The most abundantly caught species in the first study were *Andrenaminutula* (Perkins, 1914), *A.dorsata* (Kirby, 1902) and *A.nigroaenea* (Kirby, 1902). We identified 17 Andrena species within those caught, representing 31% of the assessed specimens. In terms of abundance, Andrena specimens accounted for 70% of the total catches. In the second study, the most abundant species caught were *Lasioglossummorio* (Fabricius, 1793), *Panurgusbanksianus* (Kirby, 1802) and *Lasioglossumzonulum* (Smith, 1848). Lasioglossum specimens accounted for almost 50% of the total catch, with 24 species representing 18% of the total species richness. Blue pan traps yielded 748 wild bee specimens, white traps 546 and the yellow ones 1,227. On average, per site, we found 13 species in blue pan traps, 11 in white traps and 14 in yellow ones. According to the European Red List of Wild Bees (ERL), our compilation entails eight near-threatened (NT) species (second checklist) and 28 species labelled as data deficient (DD).

## Discussion

Only nine of the 150 species identified are mentioned in the Italian Red List. They are labelled as data deficient (DD) or of least concern (LC): *Andrenahattorfiana*; *Andrenaovatula* (Kirby, 1802); *Andrenasymphyti* Schmiedeknecht, 1883; *Dufoureadentiventris*; *Dufoureainermis*; *Lasioglossumbrevicorne*; *Lasioglossumlaevigatum*; *Nomadamutica* and *Nomadavillosa*. However, according to the European Red List (ERL), these species are all considered near-threatened (NT), except for *A.sympthyti*, categorised as DD. For example, a recent European Commission report links *A.hattorfiana* decline in northern Europe to habitat loss from intensive agriculture (Michez et al. 2023). These nine species exhibit diverse behavioural, diet and habitat preference traits (second checklist), which highlights the importance of maintaining diverse and heterogeneous landscapes to support their ecological requirements and ensure the provision of critical pollination services across different ecosystems (Lázaro and Alomar 2019, Katumo et al. 2022, Zanini et al. 2024). Additionally, we found specimens of the *Andrenaovatula* [species group], which would also be considered NT by the ERL if the identification could be confirmed. However, this species is visually similar to *A.afzeliella* (Kirby, 1802) and *A.ovata* Schenk, 1853, which are common in Italy and are difficult to differentiate (Praz et al. 2022). Addressing taxonomic issues, including the classification of bees, is not the aim of this work; thus, we did not include it in the second checklist. Wild bee presence and diversity depend on seasonality, floral resources and nesting site availability, though sampling methods have inherent limitations (O'Connor et al. 2019, Hutchinson et al. 2021). While our two studies were not designed for comprehensive site monitoring, the absence of *Colletes* spp. and *Dasypoda* spp. in our pan-trap samples suggests that certain species may have been under-represented.

Taxonomy often varies significantly between regions and countries, as understanding and species classification depend heavily on local expertise, available references and regional biodiversity data. Therefore, this can result in gaps or inconsistencies in the identification and classification of species. Upon consultation of the most complete regional list of wild bees from Hellrigl (2006), we found seven species in our data-set that, in 2006, were considered "not present but anticipated to migrate to South Tyrol from neighbouring regions" (DD or LC in the ERL). Additionally, we found two species believed to be "already present but without substantiated data to confirm it" (DD or LC in the ERL). We also found 11 species absent from Hellrigl's assessment, labelled LC, NT or DD in the ERL. Here, we briefly list these species and discuss potential issues with misclassifications and historical identifications.

- Presence of *Hylaeusleptocephalus* (Morawitz, 1870), *Andrenaventralis* Imhoff, 1832 and *Andrenaalfkenella* Perkins, 1914 in South Tyrol was considered highly probable. Indeed, before us, the first two species were found in 2003 and the third one in 2006/2007 (Kopf 2008).
- As previously discussed, several specimens that proved challenging to diagnose were provisionally assigned to Andrenacf.barbareae (DNA barcoding failed to confirm the correctness of the identification). *A.barbareae* is present in Switzerland (Amiet et al. 2010) and Comba (2019) mentions its presence in Trentino Alto-Adige. However, reliable data regarding the distribution over South Tyrol remain deficient.
- *Andrenacarantonica* Pérez, 1902 is not included in the synopsis of Hellrigl, but it appears under the synonym *Andrenajacobi* Perkins, 1921. Recently, one female of this species was found by Timo Kopf during a survey in 2014 (unpublished data, "Die Blütenbesucher in den Gärten von Schloss Trauttmansdorff"; a report requested by the Castle Trauttmansdorff's gardens and the Laimburg Pfatten Agricultural and Forestry Research Center). For the species *Andrenagelriae* Van der Vecht, 1927, we could not find a prior record of this species in South Tyrol. Despite this, it is reportedly present in Italy, Austria, and Switzerland (Amiet et al. 2010, Gusenleitner et al. 2012, Comba 2019).
- *Andrenaminutuloides* (Kirby, 1802) and *Nomadasignata* Jurine 1807 were respectively considered "to be expected" and "not present" in Hellrigl's synopsis. However, both were found some years later by Timo Kopf (2008).
- *Andrenapandellei* Pérez, 1895 was expected to appear in South Tyrol according to Hellrigl (2006). Before us, it was collected by Timo Kopf in the gardens of the Castle Trauttmansdorff (Meran/Merano, unpublished reference mentioned above). Similarly, *A.symphyti* Schmiedeknecht, 1883 was found by T. Kopf in 2011 near Salurn/Salorno (unpublished data, report curated for the Office of Public Water Resources of the Autonomous Province of Bolzano/Bozen).
- According to *Hellrigl (2006)* also *Andrenasaxonica* Stöckhert, 1935 and *A.simontornyella* Noskiewicz, 1939 were species expected to be present in South Tyrol since they were already present in Italy, Austria and Switzerland (Amiet et al. 2010, Gusenleitner et al. 2012, Comba 2019). Still, we found no references in literature regarding their presence in the region.
- According to Comba (2019), *Andrenataraxaci* Giraud, 1861 is present in Trentino Alto Adige and Wood et al. (2023) mention that this species is difficult to differentiate from *A.pastellensis* Schwenninger, 2007. Neither of these species' has been reported in South Tyrol, which suggests that they may have been overlooked or were not present.
- *Halictuslangobardicus* Blüthgen, 1944 was absent from Hellrigl's list; however, one female individual was found in 2011 (although it represented a provisional identification since male material for comparison was missing) (Kopf et al. 2012). Similarly, *Hylaeusdilatatus* (Kirby, 1802) was confused and reported by Hellrigl as *Hylaeusannularis* (Kirby, 1802). The distinction between the two species was successively clarified by Notton and Dathe (2008). Comba (2019) reports the presence of *H.dilatatus* in Trentino Alto Adige.
- *Hylaeusstyriacus* Förster, 1871 and *Lasioglossumrufitarse* (Zetterstedt, 1838) were recently captured in South Tyrol in 2009 (Wilhalm and Schatz 2010).
- According to Comba M., *Lasioglossumtransitorium* (Schenck, 1868) is present in Trentino Alto Adige; however, we could not find any more detailed information on findings in South Tyrol. Additionally, *Nomadaemarginata* Morawitz, 1877, was similarly found in literature to be present in Italy and Austria (Gusenleitner et al. 2012, Comba 2019), but reportedly not in South Tyrol.
- *Lasioglossummonstrificum* is an NT species according to the ERL; a synonym is *Lasioglossumsabulosum* (Warncke, 1986) and neither of these names appears in the synopsis from Hellrigl. It should be noted that Timo Kopf found two females in 2011 near Salurn/Salorno (unpublished data, report curated for the Office of Public Water Resources of the Autonomous Province of Bolzano/Bozen). The distribution of this species remains unclear due, in part, to past confusion with *Lasioglossumsexstrigatum* (Schenk, 1870) (Cornalba et al. 2024). In any case, *L.sexstrigatum* is also rated as "not present, but expected", which suggests that they may have been overlooked in the past, misidentified or were not present in South Tyrol.

## Supplementary Material

81FEBFEA-9D92-5BBB-81C1-E9B754C3A790 10.3897/BDJ.13.e138625.suppl1 Supplementary material 1 Site information Data type ecological Brief description Table containing site coordinates, elevation, slope, aspect, seasonal mean temperatures and precipitations. File: oo_1118422.xlsx https://binary.pensoft.net/file/1118422 Sebastiano Zanini, Matteo Dainese, Timo Kopf, Lisa Obwegs, Matteo Anderle, Georg Leitinger, Ulrike Tappeiner

7C60B787-C910-5692-8CC9-BF0FE4B5B276 10.3897/BDJ.13.e138625.suppl2 Supplementary material 2 Identified species Data type Species list Brief description This table lists the species of wild bees caught in the two sampling years and provides information on sociality, nesting strategy, diet and threat status. File: oo_1239804.xlsx https://binary.pensoft.net/file/1239804 Sebastiano Zanini, Matteo Dainese, Timo Kopf, Lisa Obwegs, Matteo Anderle, Georg Leitinger, Ulrike Tappeiner

F58572A7-42E5-5864-8F55-42C9CAEAEDF2 10.3897/BDJ.13.e138625.suppl3 Supplementary material 3 Pan trap data: wild bees Data type abundance Brief description This table includes pan trap catches with abundance data on the collected wild bees. We have detailed the sampling date, number of sessions (replicates), pan trap identification number (or group), pan trap colour, specimen sex and the habitat type where the traps were placed. File: oo_1203779.xlsx https://binary.pensoft.net/file/1203779 Sebastiano Zanini, Matteo Dainese, Timo Kopf, Lisa Obwegs, Matteo Anderle, Georg Leitinger, Ulrike Tappeiner

DBED9A4B-6990-5F44-8A41-DDB7DECDCB52 10.3897/BDJ.13.e138625.suppl4 Supplementary material 4 Sampling methods and habitats description Data type Information on sampling methods and habitats description File: oo_1203780.docx https://binary.pensoft.net/file/1203780 Sebastiano Zanini, Matteo Dainese, Timo Kopf, Lisa Obwegs, Matteo Anderle, Georg Leitinger, Ulrike Tappeiner
