# Supplementary material for: New distribution records of wild bees (Hymenoptera, Apoidea) in South Tyrol (Italy): expanding the wild bee database
Source: Biodivers Data J. 2025 Feb 25;13:e138625. doi: 10.3897/BDJ.13.e138625 (PMC11880818; doi:10.3897/BDJ.13.e138625)
Supplement: Supplementary material 4 — Sampling methods and habitats description [file bdj-13-e138625-s004.docx]

**Appendix 4**

# Materials and methods

In the first study, we visited 14 apple orchards distributed along a gradient from apple-dominated surroundings to a more heterogeneous landscape matrix. Data collection occurred during the apple tree blossoming season (14th April – 3rd June 2021). Sampling comprised three rounds: early, peak, and late mass flowering, with an average interval of 8 ± 3 days between rounds. Nine white pan traps were positioned at each site to attract wild bees visiting apple flowers. Pan traps were plastic plant saucers (Geli GmbH; 500 mL, internal diameter of 14.5 cm), which were coated with white UV-reflecting colour (Spray-ColorGmbH, Sparvar Leuchtfarbe). Pan traps were arranged along three adjacent rows of trees at the centre of each orchard. Each trap was placed at 1.10 m height at the branch level and spaced 10 m apart along the row (3 m between rows and 10 m from the field margins). Pan traps were filled with water and detergent (one tablespoon of uncoloured, unscented liquid soap per 5 L of water) to reduce surface tension and were deployed for 48 hours in the field. The biological content of each trap was preserved in 70% ethanol.

The second study investigated how wild bee diversity and pollination services were influenced by elevation (coincident with a climatic gradient) and a bioindicator reflecting the local biodiversity used as a proxy for land-use intensity (Allan et al. 2014). In this study, we visited a total of twenty four sites: five apple orchards (visited previously during the first survey), five vineyards, four pastures, six meadows, two orchard meadows, and two annual crops (potato fields). Sampling was conducted between 10th May and 18th July 2022, with an average interval of 29 ± 5 days between repetitions. Pan traps were left in the field for 24 hours, filled with water and detergent, as we did in our first study. Together with white pan traps, we also used blue and yellow ones to attract a broader range of wild bee species. In every site, two sets of three UV-reflecting pan traps (same product specifics as in 2021) were placed at vegetation height. Each set consisted of a blue, a white, and a yellow pan trap placed 5 m apart. The sets were placed 10 m apart and at least 10 m from field margins.

For the bees collected during the first year, a specimen (of both sexes, if present) per species per site was prepared to be stored in an insect box. In the second year, we prepared one specimen (of both sexes if present) per species. All the other bees are labelled, temporarily stored in 70% ethanol, and checked regularly in case ethanol refilling is necessary. The specimens will be temporarily kept by the Institute for Alpine Environment at Eurac Research for further research and then gifted to the “Naturmuseum Südtirol”, the South Tyrol Museum of Nature in Bolzano/Bozen.

For each sampling site, we provide the coordinates, their unique “site code”, and the year of sampling (Suppl. material 1). We also noted elevation, slope, and aspect at the point level (coordinates) and the mean elevation and mean slope, with their standard deviations calculated within a 100m radius. We retrieved the climate data of the sites using the software ClimateEU (Marchi et al. 2020): we used estimates at a 1km resolution for 1991-2020. Thus, we recorded each location’s average maximum and minimum air temperatures and total precipitations per season (winter, autumn, spring and summer) during the 30 years of available data.

# Habitat descriptions

Here, we concisely portray the general characteristics of the type of habitat we visited in 2021 and 2022. An extensive description of the habitats surveyed within the Biodiversity Monitoring South Tyrol (BMS) is available in the “Handbook Biodiversity Monitoring South Tyrol” (Hilpold et al., 2023).

**Apple Orchard**

The trees are grafted onto a dwarfing rootstock, spaced approximately one meter apart within rows about three meters apart. These trees reach heights of two to three meters, with a planting density in South Tyrol ranging from 3,000 to 5,000 trees per hectare. (Michelini et al., 2022). These orchards are managed as organic crops (site code: OAO) or with “integrated pest management (IPM) (site code: OAC). The undergrowth is typically kept clear to facilitate harvesting and tree maintenance. However, this changes from owner to owner: some grow grass only on the inter-row spacing; some are opposite, and others keep low vegetation on the row and inter-row.


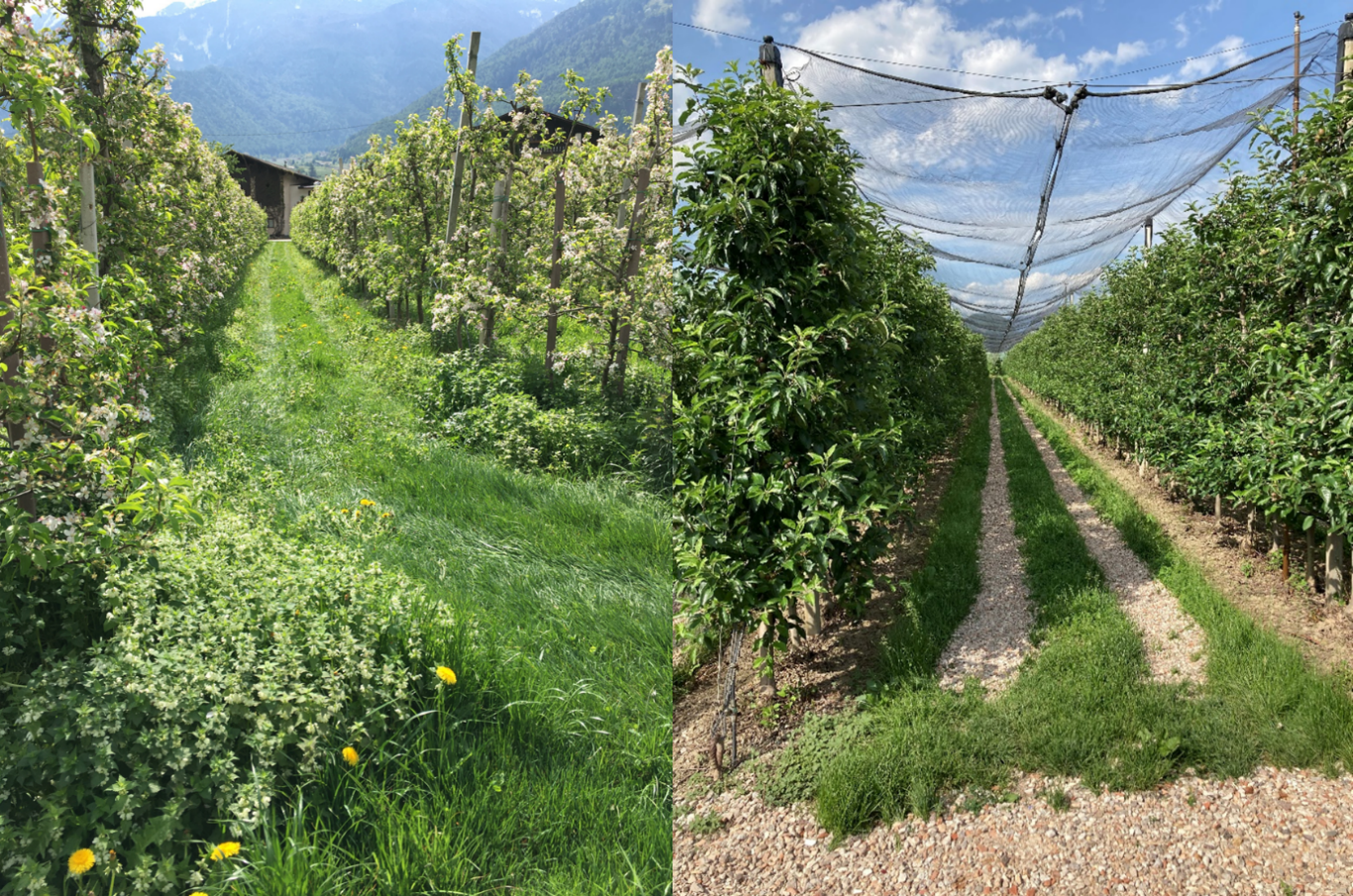


Figure 1 Intensively managed apple orchards both cultivated following IPM practices. The left side is Vinschgau/Val Venosta (site 173_OAC); the picture on the right is near Neumarkt/Egna (site 179_OAC).

**Vineyard**

These areas are usually on well-drained, sunny slopes with suitable soil types. The management practices are organic or IPM. Vineyards in South Tyrol are cultivated with grapevines typically planted in rows spaced about two to three meters apart or more, depending on the form of cultivation, with vines planted approximately one meter apart or less within each row. Vine density ranges from 3,000 to 4,500 vines per hectare (ha). Vineyards are grown in steep areas (site code: WYS) and flat areas (site code: WYF). These vineyards are managed using organic practices or integrated pest management (IPM). The inter-row spacing may be maintained with grass or low vegetation while the rows are kept clear (bare soil) or occasionally mowed. Some vineyards sow wildflower strips or leave the grassy vegetation to grow high (max 1 m) and mow alternatively every other row.


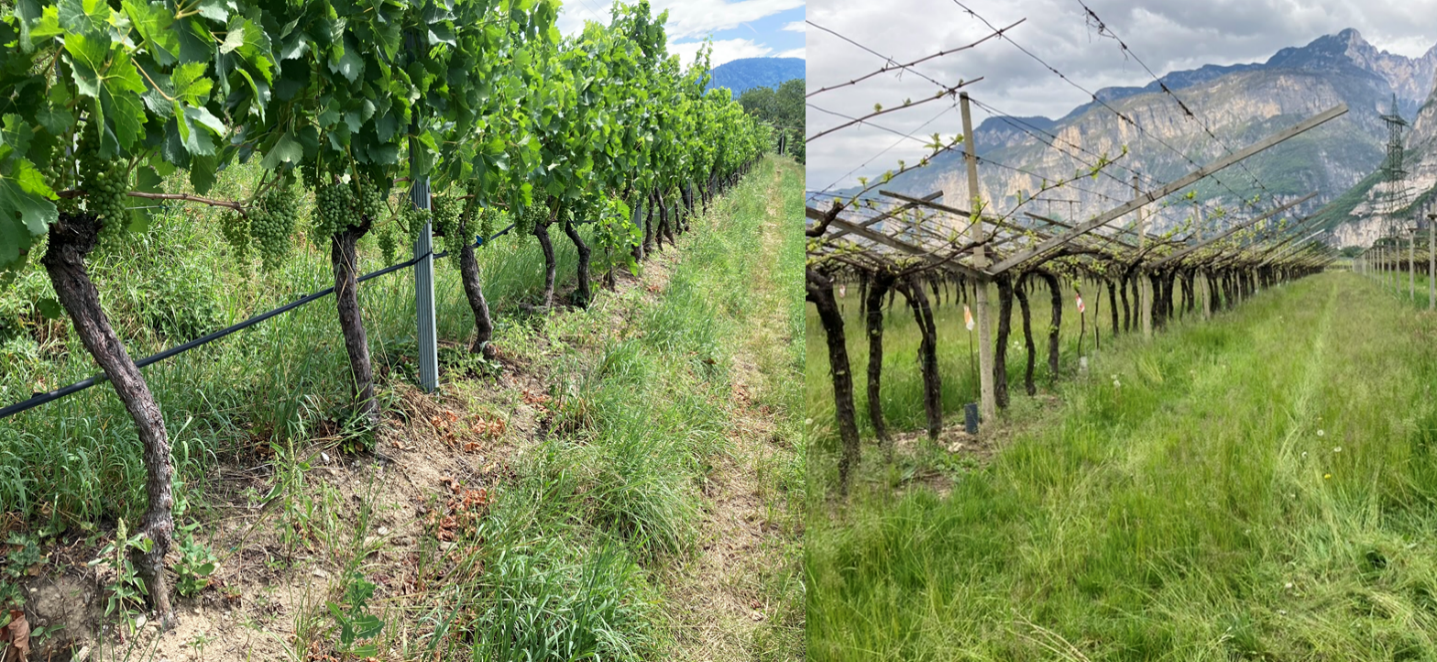


Figure 2 Vineyards cultivated near Meran/Merano (left) and Salurn/Salornoo (right) (site 202_WYS & 197_WYF). The picture on the left shows a flat surface of a series of steps in a terraced area.

**Meadow**

Meadows are a semi-natural habitat characterised by an open field of grasses, herbs, and wildflowers. They are typically found in areas with moderate to low soil fertility and may be maintained by grazing or periodic mowing to prevent succession to shrubland or forest. Meadows can vary in moisture levels, from dry upland meadows to wetter, low-lying areas. Plant density and species composition can vary greatly depending on management practices, soil type, and local climate.

The subdivision made by the BMS for the site codes HEM and HNM is shown below:

- Middle letter
  - E = subsidised meadows considered an area with “High Nature Value” (only one cut after 15 July, slightly fertilised with solid manure).
  - N = non-subsidised meadows (3 to 1 cut, good nutrient supply and early first mowing date).
- Final letter
  - M = colline-montane belt (250 – 1800 m a.s.l.).


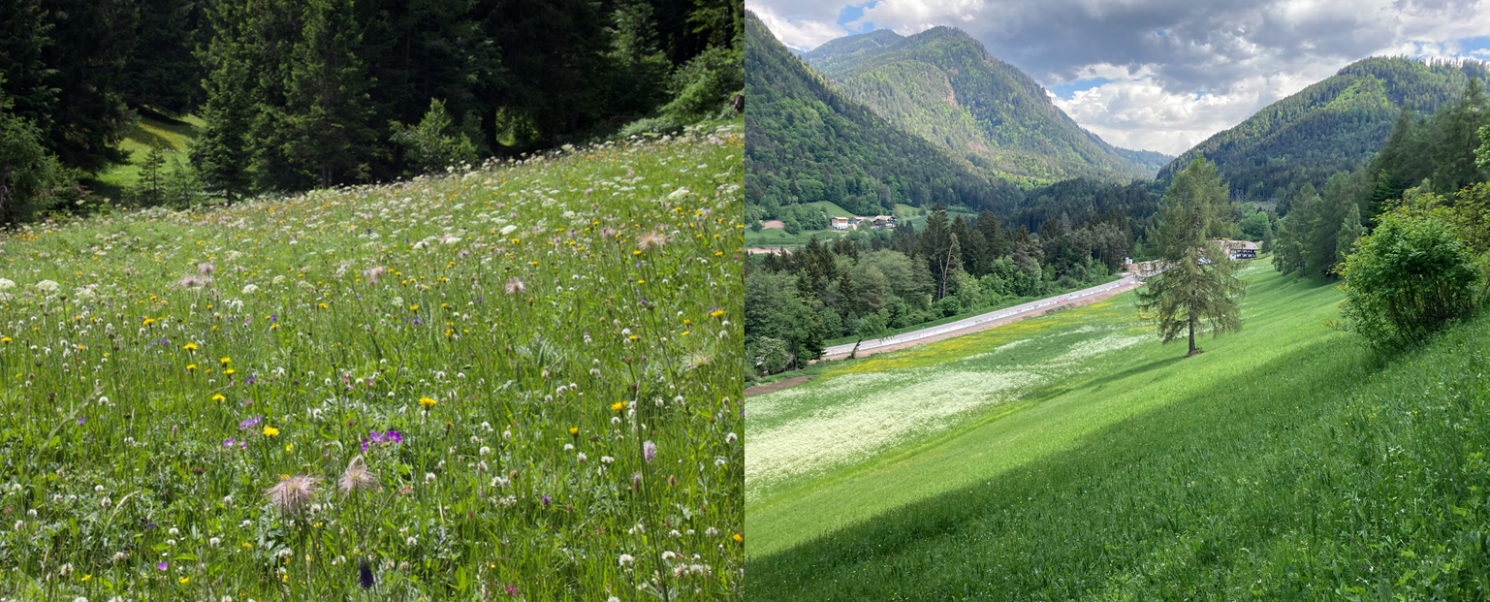


Figure 3 Two managed meadows (subsidised meadows). On the left side, the elevation is 1430 m a.s.l. (site 014_HEM) and on the right side at 928 m a.s.l. (site 020_HEM).

**Pasture**

Pasture is an agricultural habitat for livestock such as cattle, sheep, and goats. This habitat consists of grass and other forage plants that are managed to provide sustained grazing. In South Tyrol, we find permanent pastures rotationally grazed to prevent overgrazing and promote plant diversity. The density of grazing animals per hectare can vary based on the land’s carrying capacity, which the province rules. Pasture plant biodiversity can be high, particularly in less intensively managed systems. With the site code PAC, the BMS grouped pastures found in the colline-submontane zone (250 – 800 m a.s.l.); with PAM, the subalpine zone (1800 -2200 m a.s.l.).


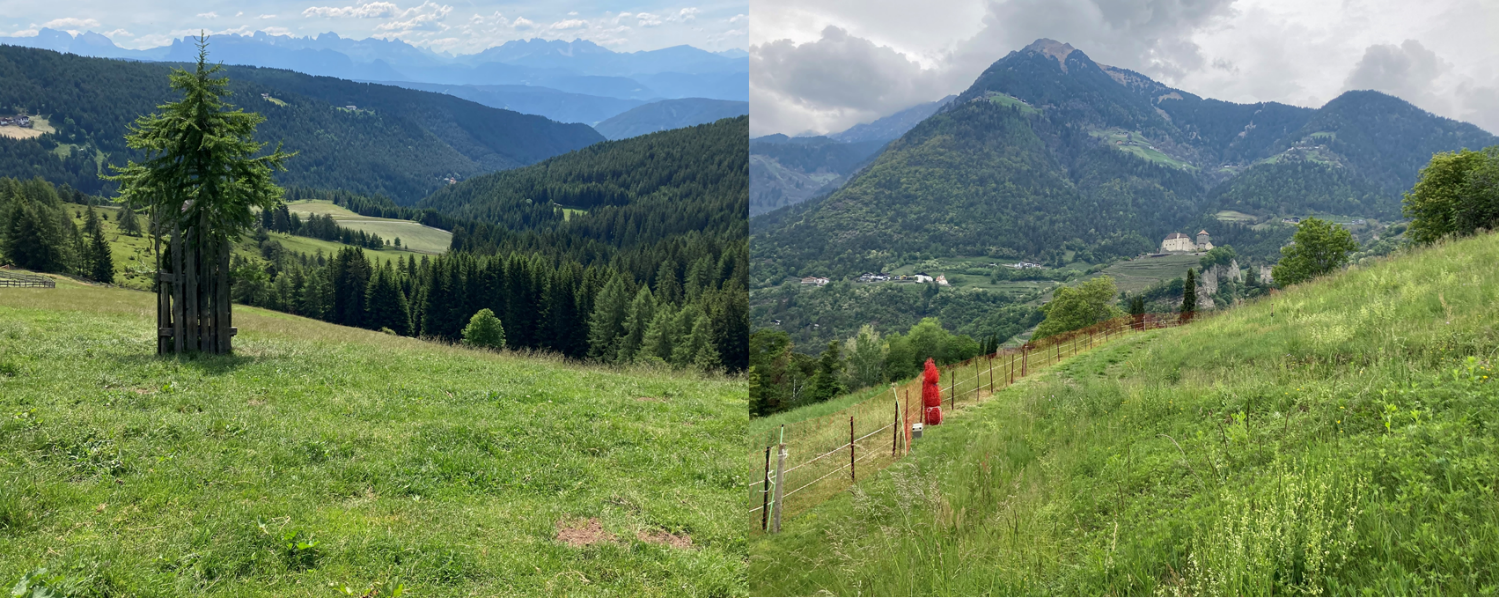


Figure 4 Two pastures outside our study sites but close to 010_HEM and 202_WYS. In the left site, cows and successively sheep graze the forage, and the elevation is 1532 m a.s.l.; in the right site, sheep and goats are the grazers, and the elevation is 440 m a.s.l.

**Orchard Meadow**

Orchard meadows are a mixed-use habitat that combines fruit trees and grassy areas, typically featuring trees planted at wider spacings of about five to eight meters apart, allowing for light penetration and undergrowth development. In regions like South Tyrol, these meadows are rare because of the low productivity and extensive use of resources compared to high-density orchards (Schönafinger,2023). The undergrowth may be mowed or grazed by livestock, maintaining an open structure. Plant species richness in the undergrowth is generally high, supporting various insects, birds, and small mammals (Guariento et al., 2020).


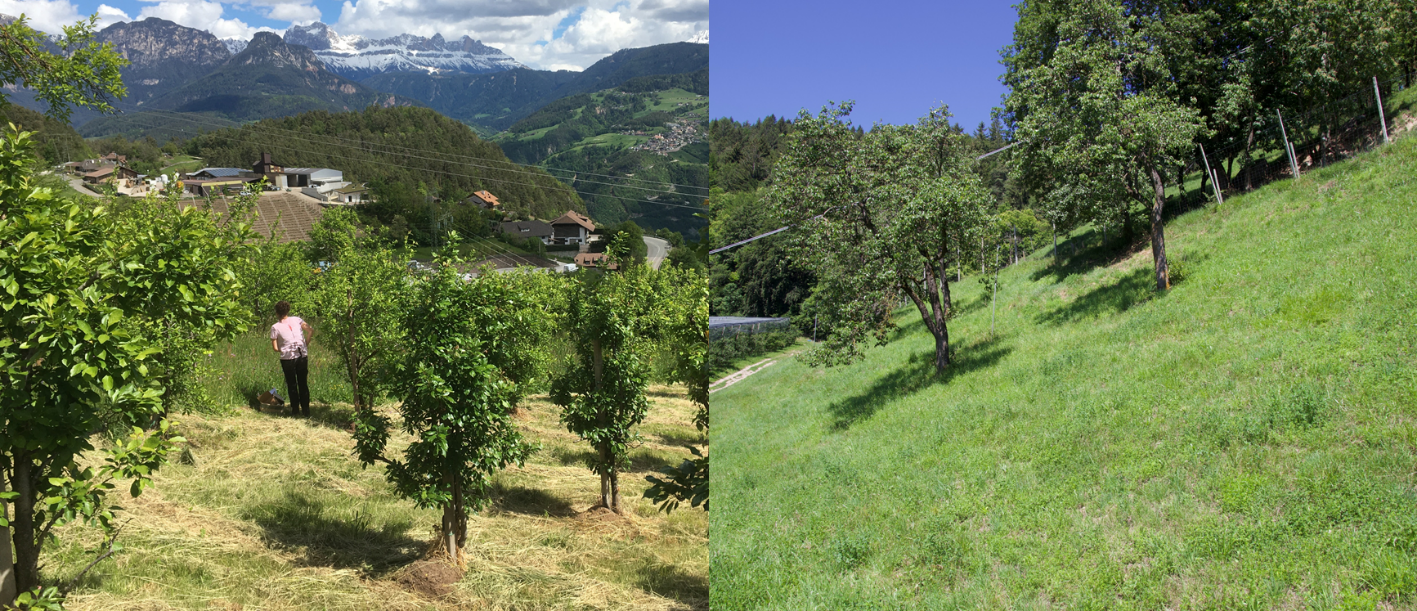


Figure 5 Traditional orchard meadows called “Streuobstwiesen” on the way to Ritten/Renon (site 346 OME); on the right picture, nearby Prissian (site 347_OME).

**Crop Field**

These fields are highly managed through ploughing, irrigation, fertilisation, and pest control. The BMS selected mainly maise fields and fields of other cereals. However, during our study, the annual crop cultivated was potato. The monoculture of potatoes shows rows of potato plants densely vegetated with bare soil inter-rows. Meadows surrounded the two fields visited for this work.


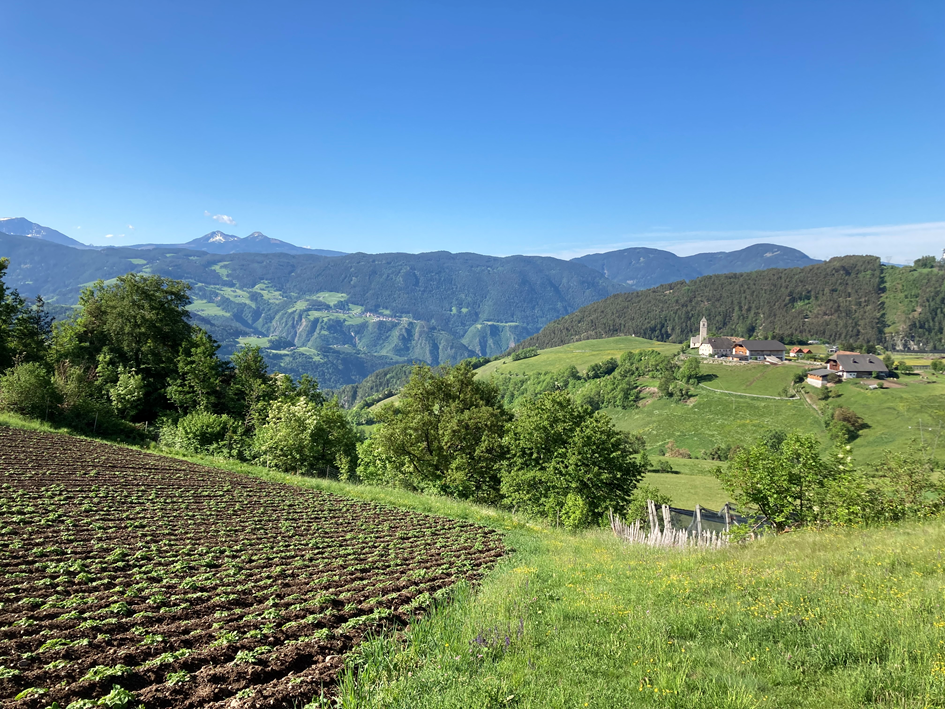


Figure 6 Potato field surrounded by a hay meadow in the Ritten/Renon area (site 155_CFC).

# Copyright of the pictures in the text:

Sebastiano Zanini took all the photos. You may use the images for non-commercial purposes only. Prior written authorisation is required for commercial use. When using the images, you must credit Sebastiano Zanini as the photographer.

# References:

Allan, E., Bossdorf, O., Dormann, C. F., Prati, D., Gossner, M. M., Tscharntke, T., ... & Fischer, M. (2014). Interannual variation in land-use intensity enhances grassland multidiversity. *Proceedings of the National Academy of Sciences*, *111*(1), 308-313. https://doi.org/10.1073/pnas.1312213111

Guariento E., Colla F., Steinwandter M., Plunger J., Tappeiner U., Seeber J., 2020; 10(6):767. Management Intensification of Hay Meadows and Fruit Orchards Alters Soil Macro- Invertebrate Communities Differently. *Agronomy*. https://doi.org/10.3390/agronomy10060767

Hilpold, A., Anderle, M., Guariento, E., Marsoner, T., Mina, M., Paniccia, C., Plunger, J., Rigo, F., Rüdisser, J., Scotti, A., Seeber, J., Steinwandter, M., Stifter, S., Strobl, J., Suárez-Muñoz, M., Vanek, M., Bottarin, R., & Tappeiner, U. (2023). Handbook Biodiversity Monitoring South Tyrol. <https://doi.org/10.57749/2QM9-FQ40>

Marchi, M., Castellanos-Acuña, D., Hamann, A. *et al.* ClimateEU, scale-free climate normals, historical time series, and future projections for Europe. *Sci Data* **7**, 428 (2020). https://doi.org/10.1038/s41597-020-00763-0

Michelini, S., Tscholl, S., Erschbamer, J., Plaikner, D., Egarter Vigl, L., & Guerra, W. (2022, 09/27). KULTIVAS: feasibility study of a variety-location model for apple cultivation. 4. https://doi.org/10.23796/LJ/2022.008

Schönafinger, A. (2023). Orchard Meadows in South Tyrol: Spatio-Temporal Development and Agro-Ecological Evaluation. <https://bibsearch.uibk.ac.at/AC16883557>
